# Role of Cortico-Cancellous Heterologous Bone in Human Periodontal Ligament Stem Cell Xeno-Free Culture Studied by Synchrotron Radiation Phase-Contrast Microtomography

**DOI:** 10.3390/ijms18020364

**Published:** 2017-02-10

**Authors:** Serena Mazzoni, Sara Mohammadi, Giuliana Tromba, Francesca Diomede, Adriano Piattelli, Oriana Trubiani, Alessandra Giuliani

**Affiliations:** 1 Department of Clinical Sciences—Unit of Biochemistry, Biology and Physics, Polytechnic University of Marche, Via Brecce Bianche 1, 60131 Ancona, Italy; s.mazzoni@alisf1.univpm.it; 2 Sincrotrone Trieste S.C.p.A., Strada Statale 14 km 163.5 in AREA Science Park, 34149 Trieste, Italy; patezian@gmail.com (S.M.); giuliana.tromba@elettra.trieste.it (G.T.); 3 Department of Medical, Oral and Biotechnological Sciences, University of Chieti-Pescara, 66100 Chieti, Italy; francesca.diomede@unich.it (F.D.); apiattelli@unich.it (A.P.); trubiani@unich.it (O.T.)

**Keywords:** periodontal ligament stem cells, phase-contrast microtomography, biomaterial, osteogenesis, xeno-free medium

## Abstract

This study was designed to quantitatively demonstrate via three-dimensional (3D) images, through the Synchrotron Radiation Phase-Contrast Microtomography (SR-PhC-MicroCT), the osteoinductive properties of a cortico-cancellous scaffold (Osteobiol Dual Block—DB) cultured with human Periodontal Ligament Stem Cells (hPDLSCs) in xeno-free media. In vitro cultures of hPDLSCs, obtained from alveolar crest and horizontal fibers of the periodontal ligament, were seeded onto DB scaffolds and cultured in xeno-free media for three weeks. 3D images were obtained by SR-PhC-microCT after one and three weeks from culture beginning. MicroCT data were successively processed with a phase-retrieval algorithm based on the Transport of Intensity Equation (TIE). The chosen experimental method, previously demonstratively applied for the 3D characterization of the same constructs in not xeno-free media, quantitatively monitored also in this case the early stages of bone formation in basal and differentiating conditions. Interestingly, it quantitatively showed in the xeno-free environment a significant acceleration of the mineralization process, regardless of the culture (basal/differentiating) medium. This work showed in 3D that the DB guides the osteogenic differentiation of hPDLSCs in xeno-free cultures, in agreement with 2D observations and functional studies previously performed by some of the authors. Indeed, here we fully proved in 3D that expanded hPDLSCs, using xeno-free media formulation, not only provide the basis for Good Manufacturing Practice (preserving the stem cells’ morphological features and their ability to differentiate into mesenchymal lineage) but have to be considered, combined to DB scaffolds, as interesting candidates for potential clinical use in new custom made tissue-engineered constructs.

## 1. Introduction

Skeletal bone diseases are nowadays a worldwide emergency. The recovery strategies following tissue damage are nowadays focused on bone regeneration rather than on repair. In this direction, the challenge with bone tissue engineering is to find a mechanically competent osteoconductive/inductive construct guaranteeing the cell attachment, the maintenance of cell properties, and their differentiation into osteogenic lineage [1].

The quantity of autologous bone is limited in a given individual [2], thus an allogenic biomaterial, showing the same structural features, could be a suitable alternative. Osteobiol^®^ Dual Block (DB) is a collagenated porcine scaffold, preserving cancellous and cortical bone structure that, for its characteristics [3,4,5], has been hypothesized to be an ideal support for bone reconstructions.

Moreover, recent literature has shown that several adult tissues contain a population of stem cells (SCs), identified in the stromal tissue, that are able to self-renew and regenerate tissues as bone, cartilage, tendon, skeleton muscle, neuron, and oral tissues [6,7]. In particular, the SC population found in human periodontal ligament (hPDL) is potentially attractive for its capacity to regenerate the tissues, exhibiting high proliferative capacity, immunomodulatory property, potential to differentiate into osteogenic, adipogenic, and chondrogenic lineages, and moreover, possessing the capacity to generate new bone after ectopic transplantation [8,9,10].

On the other hand, the tissue organization and the tissue-building process are conventionally studied through microscopy techniques, such as light, fluorescence, scanning, and transmission electron microscopy. These approaches only allow researchers to achieve just two-dimensional (2D) local information, or otherwise involve sophisticated three-dimensional (3D) reconstruction of serial sections. Moreover, the X-ray radiography presents the same limit: the images are also delivered in 2D in this case, often causing misleading interpretations of radiographs due to the superimposing of anatomical structures that can hamper the revelation of soft-to-hard tissue relationships [11]. In this context, the impact of the microtomography (microCT) technique has been revolutionary, enabling researchers to view internal sample details with unprecedented precision, high resolution, and in a non-destructive way [12]. On the other hand, despite the high reliability to calculate the different morphometric parameters in bone sites [13], the inherent characteristics of the photon beam produced by laboratory sources often prevent precise analysis of the bone mineral density and of the mineralization process itself. Third-generation synchrotron light sources may be very contributive to overcome the previous limits of laboratory microCT facilities because they produce brilliant photon beams, with spatial and temporal coherence properties at the sample stage, which are also suitable for application of phase sensitive X-ray imaging methods [14,15]. In this direction, in order to efficiently study the engineered bone during its mineralization process, the imaging quality can be enhanced through the use of phase-contrast (PhC) microCT. In fact, the phase-contrast X-ray imaging provides a substantially enhanced contrast resolution for both soft and mineralized tissues compared to conventional X-ray tube microCT experimental setups. The level of detail within the tomographic images is sufficiently consistent to allow the discrimination between the different levels of mineralization in engineered bone sites [16]. Indeed, the edges and the interfaces between collagenated and mineralized bone (or between areas at different mineralization degrees) could be clearly discernible because of the beam refraction occurring at the edges between tissues with different refraction indexes.

We reported, in a previous study, the early stages of in vitro bone formation in collagenated porcine DBs cultured with human Periodontal Ligament Stem Cells (hPDLSCs). Results were validated by synchrotron radiation (SR) X-Ray PhC-microCT [16], showing that, also at the 3D level and starting from the second week of culture, newly formed mineralized bone was detected in all the scaffolds, both in basal and differentiating media. The limit of this demonstrative research was linked to the nature of the used (not xeno-free) media; it was not clear if they provided equal or increased performance (in cell proliferation, protein production, etc.) over serum, precluding the development of stem cell clinical treatments meeting the regulatory guidelines and having a cost-effective system compatible with their end-use.

Moreover, some of the authors recently reported that expanded hPDLSCs, using xeno-free media formulations, provide the basis for Good Manufacturing Practice (GMP). They preserve: (1) the stem cells’ morphological features and karyotype; (2) the expression of stemness and pluripotency markers; and (3) the ability to differentiate into mesenchymal lineage [7]. The same authors also monitored the morphological features of hPDLSCs cultured in xeno-free media in the presence of DB scaffolds, checking their ability to induce the differentiation process [17]. The analysis suggested that, after one week of culture, both uninduced cells and cells induced to osteogenic differentiation joined and grew on DB secreting extracellular matrix that, in osteogenic induced samples, was hierarchically assembled in fibrils. Furthermore, functional studies suggested that the biomaterial could drive the osteogenic differentiation process of hPDLSCs: indeed, they showed a significant increased response of calcium transients in DB-based constructs, both with undifferentiated and differentiated cells stimulated with calcitonin and parathormone. However, the limit of these last investigations was linked to the 2D nature of the characterization. Therefore, before translating the previous finding into clinical research, it was necessary not only to avoid variability introduced by non-xeno-free cultures [18], but also to validate the protocols by the quantitative data extracted from 3D analysis.

For this reason, in the present study, the early stages of in vitro bone formation in collagenated porcine cortico-cancellous DBs cultured with human peridontal ligament stem cells (hPDLSCs) in xeno-free media were investigated by synchrotron radiation-based phase-contrast microtomography (SR-PhC-microCT). This study provided, for the first time to the authors’ knowledge, fundamental quantitative information, at the 3D level, on the kinetics of these processes in xeno-free media, quantitatively evaluating and discussing mismatches with previous findings in not xeno-free media. This is clearly a fundamental step in order to speed up the therapeutic use of these constructs in clinical practice, avoiding variability and possible misinterpretations of not xeno-free cultures.

## 2. Results

**Figure 1:**
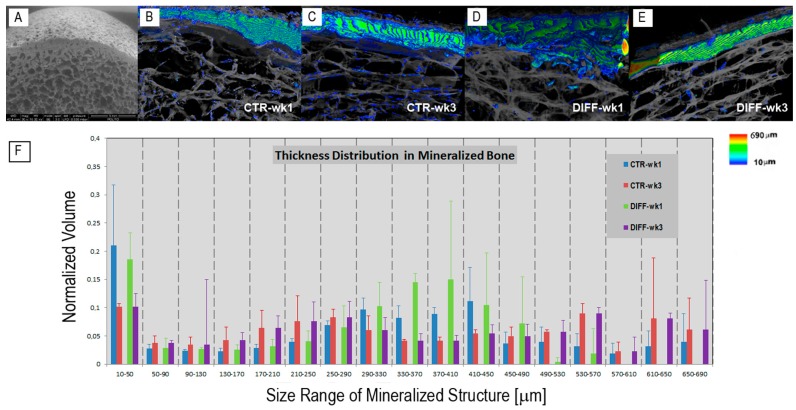
(**A**) Scanning electron microscopy image of the Osteobiol^®^ Dual Block before cell seeding: the cortical bone is anchored to cancellous bone, mimicking the natural human bone architecture; (**B**–**E**) Dual Block cultured with human Periodontal Ligament Stem Cells in xeno-free media. Synchrotron Radiation Phase-Contrast Microtomography (SR-PhC-microCT) three-dimensional (3D) images of sampling sub-volumes in (**B**,**C**) basal; and (**D**,**E**) osteogenic conditions. The cross-talk between cells, media, and scaffold produced 3D microCT images with two different phases, corresponding to different δ (refractive index decrement) values: the phase corresponding to demineralized Dual Block (DB) scaffolds (rendered in translucent gray) and the phase due to the contrast produced by the newly formed mineralized bone (colored in agreement to the color map of bone thickness distribution on the right); (**B**,**D**) at week 1; (**C**,**E**) at week 3; (**F**) Histograms of the fully mineralized bone thickness distribution in the trabecular portion of the investigated samples at weeks 1 and 3 of culture.

The rationale that collagenated DBs cultured with hPDLSCs in xeno-free media could drive the osteogenic differentiation process of the cells was shown for the first time by some of the authors using transmission electron microscopy (TEM), qRT-PCR, and functional studies [17]. However, the osteogenic differentiation processes, when conventionally investigated by microscopy techniques (such as TEM), often deliver a not fully reliable quantitative analysis, due to the characterization technique limited to 2D local information. Therefore, we validated the previous culture protocols by the quantitative data extracted from 3D analysis. Indeed, SR-PhC-microCT was able to easily discriminate the newly formed mineralized phase from the demineralized scaffold of porcine origin. The DB scaffold before cell seeding is shown in Figure 1A by scanning electron microscopy.

The cross-talk between cells, xeno-free media, and DBs modified the scaffold structure and density over three weeks of time from culture starting, producing 3D microCT images in which two different phases—the demineralized scaffold and the newly mineralized bone—with different refractive indexes were present. Representative samples, cultured both in basal (CTR) and differentiating (DIFF) media for one and three weeks from cell seeding, are shown in Figure 1B–E. The newly formed mineralized phase and the scaffold were virtually colored to facilitate their discrimination in 3D. The newly formed phase was depicted in graded RGB colors, corresponding to a color map (Figure 1, right) proportional to the structure thickness (scale: 10–690 μm), and the scaffold matrix was shown in translucent grey to better visualize the 3D interconnection with the mineralized bone phase.

MicroCT revealed that a significant (detectable) quantity of the newly formed phase was present already after the first week in both basal and differentiating media, and that this phase occurred preferentially in the compact areas. This finding mismatches with previous results obtained on not xeno-free media [16], where the mineralized clusters were detectable just starting from the second week of culture in both the culture media, and were mainly concentrated in the trabecular portion.

In the present study, the amount of the newly formed mineralized bone was calculated, exclusively in the cancellous areas, by counting the corresponding voxels underlying the peak associated with the relevant phase. The obtained data were expressed as mBV/BV (%) (i.e., the ratio of the newly formed mineralized volume (mBV) to the total scaffold volume (BV)). The mean trabecular thickness and the mean mineralized bone thickness were also evaluated in the same cancellous areas, expressed as BTh (μm) and mBTh (μm), respectively. These morphometric data were reported in Table 1. Interestingly, a comparison between the xeno-free cultures in basal medium (CTR) and in differentiating medium (DIFF) showed, within the limits of the sample size, that no significant differences (*p* > 0.05) were present in mBV/BV ratios between CTR and DIFF cultures at identical time points. However, highly significant (*p* < 0.001) differences were detected between the first and the third week of culture in both the culture media. The same behavior was also observed in the evaluation of the mean trabecular thickness, with an increment of almost 50% of the thickness from the first to the third week of culture in both the culture media. In contrast, no significant differences (*p* > 0.05) were present considering the mean mineralized bone thickness parameter, neither in time for the same medium, nor between CTR and DIFF cultures at identical time points.

On the other hand, a quantitative analysis based solely on the comparison of the average morphometric values, like the previous one (based on Table 1 data), is often not complete, requiring a more effective study of the mineralized bone size distribution. For this reason, to investigate more deeply the changes over time of the newly formed bone, the “mineralized bone thickness distribution vs. the bone volume normalized to the total sample volume” was also assessed in the trabecular portion of the samples. The histograms of the mineralized bone thickness distribution in all the investigated samples were reported in Figure 1F. It was shown that, while after the first week of culture the distribution was bimodal for both the media, with small and medium mineralized nuclei, respectively 10–50 and ~370 μm thick in diameter, after the third week of culture the distribution was trimodal for both the media, with small, medium, and large mineralized nuclei, respectively 10–50, 250–290, and 550–650 μm thick in diameter. The presence, after three weeks, of big mineralized nuclei, with diameter of ~600 μm, justifies the overall evaluation of the mean bone thickness, with its highly significant increment from the first to the third week of culture in both the culture media.

**Figure 2:**
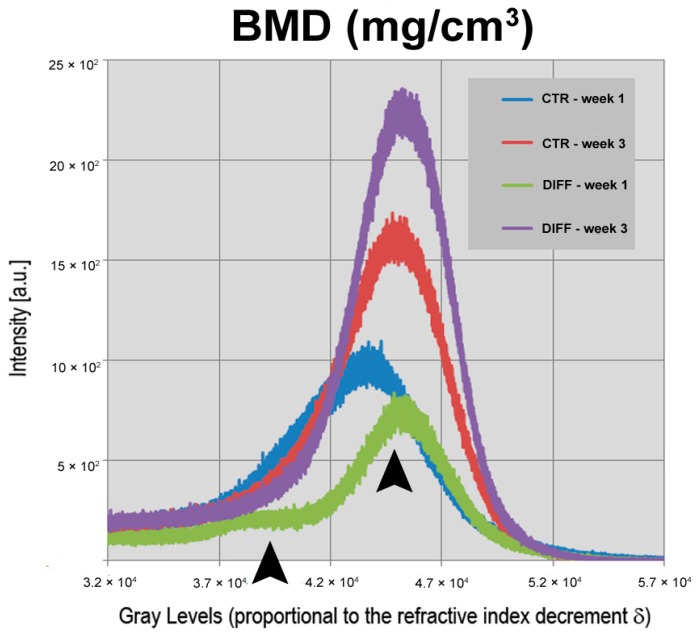
Portion of the “intensity counts vs. gray levels” profiles. The grey levels are proportional to the refractive index decrement δ, nearly proportional to the physical density ρ of the newly formed mineralized phase. The integrated areas of the represented peaks correspond to the volumetric amount of the newly formed mineralized bone in scaffolds cultured in basal and differentiating xeno-free media. The two black arrowheads show the two different levels of mineralization after the first week of culture.

Furthermore, we tested another relevant quantitative parameter, the growth kinetic of the physical density ρ (mg/cm^3^) of the newly formed mineralized phase. This physical datum, often referred to “Bone Mineral Density (BMD)” in the literature, was shown to increase from the first to the third week, both in basal (CTR) and in differentiating (DIFF) medium. This was demonstrated by studying the profiles of the “Intensity Counts vs. Grey Level” (Figure 2). Indeed, due to the experimental phase-contrast set-up and the TIE algorithm implemented for the data analysis, the grey levels—here referred to an unsigned 16-bit scale—are proportional to the refractive index decrement δ, that in turn is nearly proportional to the physical density ρ (mg/cm^3^) of the newly formed mineralized phase.

The ρ values found in all the samples were compatible, at the same experimental conditions, with those related to fully mineralized bone (calibration data not shown). On the other hand, evidence obtained by light and electron microscopy [17] had already confirmed that the newly formed phase was mineralized bone. Interestingly, a comparison between the xeno-free cultures showed that no significant differences (*p* > 0.05) were present in terms of BMD between CTR and DIFF cultures at identical time points, with two mineralization levels after the first week of culture (first level: mean grey value *x* ≅ 4.0 × 10^4^; second level: mean grey value *x* ≅ 4.5 × 10^4^) and a single mineralization level after three weeks of culture (mean grey value *x* ≅ 4.5 × 10^4^), as shown by the two black arrowheads in Figure 2.

## 3. Discussion

We started from the paradigms described in the introduction and reasoned from the promising results obtained with conventional 2D approaches on hPDLSCs isolated from periodontium biopsy and seeded on cortico-cancellous DB scaffolds in xeno-free media [17]. In this study, we presented the use of Synchrotron Radiation-based phase-contrast MicroCT to quantitatively investigate in 3D, for the first time to the authors’ knowledge, the kinetics of the early stages of the mineralization process in the same constructs.

This analysis disclosed bone deposits, in the shape of spots or fibrils (as shown in Figure 1B–E), detected already from the first week of culture in both basal and differentiating media.

All the investigated morphometric parameters, within the spongy volume of the samples, showed that no significant differences were present between CTR and DIFF cultures at identical time-points, but that a significant increase of the mineralized volume and of the overall mean trabecular thickness were detected between the first and the third week of culture in both the culture media.

In our previous microCT experiments [16], performed on DB-based constructs not cultured in xeno-free media, we did not observe any signal of mineralization after the first week. The comparison of the two scenarios indicates the possible acceleration of the process in xeno-free conditions, especially in the basal medium where the cells should take a longer time to differentiate than in differentiating medium. Indeed, it was demonstrated in a previous study [7] that cells cultured under xeno-free conditions showed higher levels of mineralization at precocious time than cells maintained under not xeno-free conditions. We hypothesize that the osteogenic process could be enhanced by the molecular and chemical composition of the xeno-free medium. Indeed, in the present and in the previous studies [17], we used chemically defined media (License protected under Lonza patent; MSCBM-CD, Lonza) in order to get standardized culture protocols and to avoid concerns related to fetal bovine serum (FBS) manipulation and highly different composition between lots.

Interestingly, SR-PhC-microCT studies revealed an overall significant trabecular thickness increase in both media, from the first to the third week of culture, while only a slight increase (not significant) was observed in terms of thickness of the mineralized portion. This seems to indicate that the scaffold bioresorption, which was previously proven to be more accentuated up to the second week of culture [16], is well compensated by the cells growth. As shown by cell viability (MTT) assay, TEM, and Quantitative Real-Time PCR [17], the present studies fully confirmed in 3D that the biomaterial does not interfere with cell growth, suggesting also its possible specific role in osteogenic induction of the undifferentiated hPDLSCs, activating genes including *BMP-2/4*, *RUNX-2*, and *Collagen1A1*. In fact, another relevant quantitative result of this work was the distribution of the physical density of the mineralized clusters and its modification from the first to the third week, both in CTR and in DIFF media, as shown by the Figure 2. Indeed, while at the very early stage (first week)—as confirmed by functional studies [17]—these cultures were heterogeneous, with the extracellular matrix at different levels of mineralization, whereas after three weeks of culture in xeno-free media the mineralization process appeared almost complete, at least based upon considering the values achieved by the physical density of the mineralized bone (BMD—mg/cm^3^).

## 4. Materials and Methods

### 4.1. Scaffold Material

The scaffold, named OsteoBiol^®^ Dual Block (Tecnoss^®^ Dental, Coazze, Italy), is a collagenated block constituted by natural cancellous and cortical porcine bone. The cortical bone is naturally anchored to cancellous bone in order to provide stability after grafting. This scaffold guarantees, due to its rigid consistency, that the original volume of grafting site can be preserved and it is indicated for horizontal crest reconstructions. This biomaterial was selected for the previously mentioned peculiar features; in particular, because it possesses a cortico-cancellous structure similar to human bone [16,17].

Small blocks (20 × 15 mm^2^) were cut into slices with thickness of approximately 5 mm using a Buehler low-speed saw equipped with a diamond water blade (Buehler Isomet, Lake Bluff, IL, USA). The slices were processed through washes in distilled water (d-H_2_O), sonicated for 1′ (1 cycle 70 W, SONOPULS, HD2070, Bandelin; Berlin, Germany), and then further washed with sterile PBS (LiStarFish, Milan, Italy). After that, the scaffolds were sterilized using UV irradiation overnight before use.

### 4.2. Isolation and Culture of Periodontal Ligament Stem Cells

The Medical Ethics Committee at the Medical School (“G. d’Annunzio” University, Chieti, Italy) obtained written approval (n. 266/17.04.14, Principal Investigator: Trubiani Oriana) for the human periodontal ligament collection, and written informed consent for clinical research and for the processing of personal data from each participant (range age: 20–35 years). The Department of Medical, Oral and Biotechnological Sciences and the Laboratory of Stem Cells and Regenerative Medicine are certified according to the quality standard ISO 9001:2008 RINA (certificate No. 32031/15/S).

Isolation and culture of periodontal ligament stem cells were performed in agreement with the protocol described in Diomede et al. [17]. Briefly, periodontal ligament biopsies from five different patients were obtained from human premolar teeth, scheduled to be removed for orthodontic purposes on healthy volunteers; afterwards, the donors were de-identified.

Ex vivo expanded hPDLSCs were seeded at 1 × 10^3^ cells/well in triplicate for each group of study using a 96-well flat bottom plate. They were cultured in TheraPEAK™MSCGM-CD™ BulletKit serum free, chemically defined (MSCGM-CD) (Lonza, Basel, Switzerland) control medium (CTR) for the growth of human Mesenchymal Stem Cells (MSCs), and in MSCGM-CD medium with the addition of osteogenic supplements (DIFF) in the presence of the OsteoBiol^®^ Dual-Block (DB) scaffold for one and three weeks of time.

### 4.3. Synchrotron Radiation X-ray Phase-Contrast Microtomography

The tomographic experiments were performed at the SYRMEP beamline of the ELETTRA Synchrotron Light Source (Trieste, Italy).

The samples were investigated using the following experimental parameters: isometric voxel with edge size of 9 µm; beam energy of 16 keV; sample-detector distance of 150 mm; exposure time of 1 s per 1200 projections.

The approach was based on a phase-contrast set-up. Briefly, the effect of the X-ray beam going through the sample is described by the refractive index, *n*(r) = 1 − δ(r) + *i*β(r), where δ is the refractive index decrement and β is the attenuation index. As δ is much larger than the imaginary part β in demineralized samples like DBs, the phase-contrast microCT set-up provides greater sensitivity than the conventional absorption approach. Given that δ is proportional to the mean electron density, which in turn is nearly proportional to the physical density ρ (mg/cm^3^), by SR-PhC-microCT it was possible to monitor the mineralization evolution in the three weeks of culture in xeno-free conditions. Furthermore, due to the smoothly varying density of the constructs, occurring at the early stages of bone formation, the distributions of the real and imaginary parts of the refractive index can be considered proportional to each other (i.e., δ(r) = *ε∙*β(r)). The ε value was set to 200 after several explorative tests.

In this study, the X-TRACT software (CSIRO Mathematical and Information Science, Canberra, Australia) was used to reconstruct the 2D slices, with the application of the phase retrieval algorithm based on the Transport of Intensity Equation (TIE) [19,20] and the successive processing by the common filtered back-projection algorithm.

The commercial software VG STUDIO MAX 1.2 (Volume Graphics, Heidelberg, Germany) was applied for the 3D reconstruction of the samples and to perform the structural analysis of the trabecular portion; the mixture modeling algorithm (NIH ImageJ Plugin, https://imagej.nih.gov/ij/plugins/) was used to threshold the “intensity counts vs. gray levels” histograms.

### 4.4. Statistical Analysis

Statistical analysis was performed by one-way-ANOVA tests, using the SigmaStat 3.5 software (Systat Software, San Jose, CA, USA). All pairwise multiple comparisons were performed by the Holm-Sidak method, considering a *p*-value < 0.05 to be statistically significant.

## 5. Conclusions

In conclusion, our experimental protocols, performed at single sample-detector distance with a phase-contrast set-up and subsequent application of a phase retrieval algorithm, were proven to successfully monitor and quantify in 3D the early stages of the osteogenic process of Dual Blocks cultured with Periodontal Ligament Stem Cells in xeno-free media.

The role of DBs in guiding the osteogenic differentiation of hPDLSCs was here demonstrated in xeno-free cultures, thereby helping to reduce concerns regarding PDLSC applications [18] and encouraging further promising preclinical and clinical studies.

The feasibility and reliability of synchrotron radiation phase-contrast micro-CT analysis to study in vitro the new bone formation on collagenated bioscaffolds was here confirmed, with significant quantitative 3D information on the mineralization process kinetics in xeno-free media, which was not previously available with other conventional forms of analysis.

However, it needs to be stressed that the SR-PhC-MicroCT measurements, performed in the area of tissue engineering and biomaterials, like in the present study, have focused, to date, on in vitro or ex vivo sample characterization. Future studies need to focus on the development of these techniques toward the non-invasive imaging of engineered tissues in bioreactors and in vivo. Thus, further progress of the PhC-microCT technology will focus on translation of SR-PhC-based techniques, restricted to the limited number of Synchrotron users, to the implementation of PhC-imaging techniques in the available benchtop X-ray tubes, which are more easily available in the lab and clinical settings.

## Figures and Tables

**Table 1:** Quantitative analysis of 3D data after the in vitro tests. The whole trabecular portion of the samples was considered.

Morphometric Parameters	CTR-Week 1	CTR-Week 3	DIFF-Week 1	DIFF-Week 3	Significance
mBV/BV (%)	60.9 ± 5.5	78.9 ± 1.5	60.7 ± 4.9	78.8 ± 0.7	CTR-week 3 vs. CTR-week 1 (*p* < 0.001)
					DIFF-week 3 vs. DIFF-week 1 (*p* < 0.001)
BTh (μm)	64.7 ± 10.9	96.7 ± 0.5	68.7 ± 5.1	102.9 ± 5.6	CTR-week 3 vs. CTR-week 1 (*p* < 0.001)
					DIFF-week 3 vs. DIFF-week 1 (*p* < 0.001)
mBTh (μm)	174.6 ± 13.0	178.2 ± 34.5	172.7 ± 13.1	208.7 ± 34.1	-

mBV/BV = volume ratio of the mineralized bone structure (mBV) volume to the overall bone volume (BV); BTh = mean trabecular bone thickness; mBTh = mean trabecular mineralized bone thickness.
